# Efficient Closed Loop Simulation of Do-It-Yourself Artificial Pancreas Systems

**DOI:** 10.1177/19322968211032249

**Published:** 2021-07-30

**Authors:** Jana Schmitzer, Carolin Strobel, Ronald Blechschmidt, Adrian Tappe, Heiko Peuscher

**Affiliations:** 1 Institute for Medical Engineering and Mechatronics, Ulm University of Applied Sciences, Ulm, Germany; 2 AndroidAPS.org, Software Development, Linz, Austria

**Keywords:** type 1 diabetes, AndroidAPS, artificial pancreas, in silico trial, hybrid closed-loop, glycemic control

## Abstract

**Background:**

Numerical simulations, also referred to as in silico trials, are nowadays the first step toward approval of new artificial pancreas (AP) systems. One suitable tool to run such simulations is the UVA/Padova Type 1 Diabetes Metabolic Simulator (T1DMS). It was used by Toffanin et al. to provide data about safety and efficacy of AndroidAPS, one of the most wide-spread do-it-yourself AP systems. However, the setup suffered from slow simulation speed. The objective of this work is to speed up simulation by implementing the algorithm directly in MATLAB^®^/Simulink^®^.

**Method:**

Firstly, AndroidAPS is re-implemented in MATLAB^®^ and verified. Then, the function is incorporated into T1DMS. To evaluate the new setup, a scenario covering 2 days in real time is run for 30 virtual patients. The results are compared to those presented in the literature.

**Results:**

Unit tests and integration tests proved the equivalence of the new implementation and the original AndroidAPS code. Simulation of the scenario required approximately 15 minutes, corresponding to a speed-up factor of roughly 1000 with respect to real time. The results closely resemble those presented by Toffanin et al. Discrepancies were to be expected because a different virtual population was considered. Also, some parameters could not be extracted from and harmonized with the original setup.

**Conclusions:**

The new implementation facilitates extensive in silico trials of AndroidAPS due to the significant reduction of runtime. This provides a cheap and fast means to test new versions of the algorithm before they are shared with the community.

## Introduction

In 2013, the #WeAreNotWaiting community was founded with the objective of reducing the burden of type 1 diabetes (T1D) on patients with diabetes (PWD) and their families, especially at night.^1,2^ In 2015, these efforts resulted in the OpenAPS project, an open-source, hybrid closed-loop (HCL), do-it-yourself (DIY) artificial pancreas system (APS).^3,4^ Due to its DIY nature, OpenAPS is not commercially available, but must be built and setup individually by anyone who wants to use it. It is therefore neither regulated by the U.S. Food and Drug Administration (FDA) nor any other regulatory authority. Unlike conventional pump therapy, it uses a feedback controller to automatically regulate the subcutaneous insulin delivery of an insulin pump. For this purpose, the algorithm evaluates the measured blood glucose values obtained from a continuous glucose monitoring (CGM) sensor. Thus, the system ensures that an appropriate dose of basal insulin is administered at all times. Bolus insulin is still delivered manually by the user as needed, for example before meals.^4^ Originally, the control algorithm of OpenAPS was designed to run on a dedicated microcomputer such as an Intel Edison or Raspberry Pi.^5^ More recently, the smartphone apps AndroidAPS and Loop emerged to provide the functionality of OpenAPS on customary smartphones operated by Android and iOS, respectively.^6,7^

### Motives for Using Do-It-Yourself Artificial Pancreas Systems

Although first professional HCL systems like MiniMed^TM^ 670G and 770G (both Medtronic, Northridge, California) have been commercially available for about three years in many countries,^8,9^ many PWD still opt for DIY APS. In February 2021, the number of known users worldwide using DIY APS exceeded 2200.^10^ The estimated number of unrecorded cases is even much higher.^11^ One reason for the preferred usage of DIY APS is dissatisfaction with the usability of the 670G system, for instance, due to the need to switch to manual mode on sick days.^12^ But what convinces users even more is a community that offers globally comprehensive support and that DIY APS are cheaper.^13^ They also opt for them, as they have more customizable settings and offer more flexibility,^14^ or, plainly, because the local health care system offers no alternatives.

### Factors Limiting Greater Spread of Do-It-Yourself Artificial Pancreas Systems

But there are also disadvantages of using DIY APS. First, they are not approved by any regulatory authority and are used at one’s own risk of possible physical harm.^4-7^ Therefore, many healthcare professionals treating PWD using DIY APS find themselves in a challenging dilemma, both from an ethical and legal point of view, since the responsibilities in the case of an adverse event are not clearly defined.^15-17^ Secondly, the need to set-up the DIY APS manually may discourage patients without advanced computer skills. Furthermore, purchasing a suitable insulin pump can be challenging because of the fact that insulin pumps are typically protected against unauthorized access from third-party devices. Therefore, in many countries, there is only a limited number of compatible pumps available on the market, many of which are out of warrantee.^3^ However, current efforts to promote interoperability, for example, the Open Protocols Initiative of the JDRF, might help mitigate these shortages in the future.^18^ The fact that the DIY APS do not pass a regulatory approval process has the additional disadvantage, that the devices and algorithms are not tested in clinical trials before they are put into operation. Indeed, a number of studies based on real-world experiences and CGM data provided by users have shown, that the DIY APS algorithms keep blood glucose (BG) values stable, especially at night; also, participants reported a significant increase in quality of life.^19-28^ Nevertheless, more data obtained in randomized controlled trials is needed to support the strength of evidence. Another drawback of missing approval procedures is that the algorithms are not developed and tested following standardized procedures and quality management guidelines, that is, new versions or features always carry the risk of potentially dangerous errors.^17^

### Testing and Simulation

Software testing typically includes unit and integration tests to verify correct behavior of individual parts of the code (eg, functions) and their interfaces, respectively. However, these tests are not sufficient to examine the effectiveness and safety of the entire algorithm on a system level, that is, its interaction with a patient’s metabolism as part of a closed control loop. Such an analysis requires numerical computer simulations, also called in silico trials in the context of APS.

### The UVA/Padova Type 1 Diabetes Metabolic Simulator

**Figure 1.**
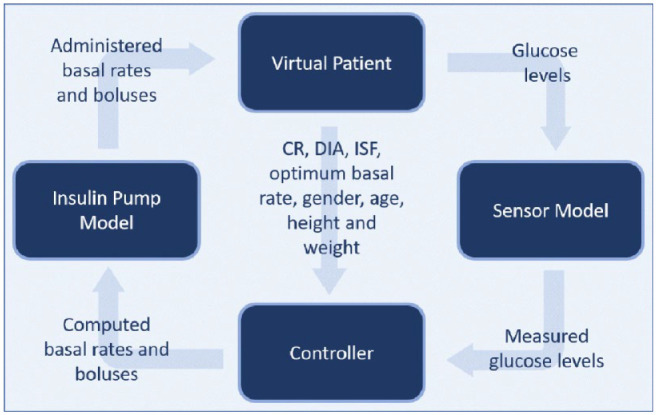
The four main components of the T1DMS with their respective inputs and outputs. Abbreviations: CR, carb ratio; DIA, duration of insulin activity; ISF, insulin sensitivity factor.

*In silico* trials of glycemic control algorithms can be conducted with the UVA/Padova Type 1 Diabetes Metabolic Simulator (T1DMS, version 3.2, The Epsilon Group, Charlottesville, Virginia),^29^ which has been approved by FDA in 2008 to replace animal trials. It offers the possibility to test APS algorithms quickly, cheaply and without endangering humans.^30^ T1DMS uses the numeric computing environment MATLAB^®^ (The MathWorks, Natick, Massachusetts), or more precisely speaking, its graphical simulation plug-in Simulink^®^.^31^ It is by default equipped with a virtual population of 10 adults, 10 adolescents (ages 13-18) and 10 children (ages 2-12),^32^ whose basic characteristics are described in Kovatchev et al.^30^ The user can configure custom scenarios to define the meals (in terms of ingested carbohydrates (CHO)) with their respective point of time the virtual patients ingest. A schematic of T1DMS is depicted in Figure 1 to illustrate the interaction between virtual patient and controller through insulin pump and CGM sensor. The interior of the controller block can be implemented by the user to model the behavior of the APS algorithm under examination.

### State of the Art

In 2020, Toffanin et al. used the T1DMS to conduct an in silico trial on the efficacy and safety of AndroidAPS.^33^ However, the authors did not implement the APS algorithm directly in the controller block, but used AndroidStudio to emulate an Android Phone and run the algorithm through the actual AndroidAPS app; also, they interfaced it with the T1DMS. Unfortunately, the procedure was only slightly faster than real time, so that several hours were needed to simulate one day of a virtual patient. This severely impairs extensive in silico trials with many virtual subjects over a longer simulation period.

### Objectives of This Work

The objective of this work is to provide a more efficient way to run simulations of AndroidAPS in T1DMS. To this end, the algorithm is re-implemented in the MATLAB^®^/Simulink^®^-based simulator rather than emulated in its original form. This requires extra care to make sure, that the results of the new implementation are identical to that of the original software. However, the new approach makes much better use of the numerical capabilities provided by MATLAB^®^ and allows for massive speed-up due to optimized and parallelized code execution. This is an important step toward systematic and automated testing of new versions or features of AndroidAPS, so that new releases can be made available to the community at far lower risk.

## Methods

### The AndroidAPS Algorithm

In the following, we provide an overview of the main components of AndroidAPS. Its centerpiece is the oref0 algorithm from OpenAPS, which calculates insulin doses following the same basic mathematics as a PWD.^4^ Supplemental features include auto-sensitivity mode (Autosens), super micro bolus (SMB) and unannounced meals (UAM).^6^ Autosens analyzes the glucose control of the past hours and adjusts the insulin sensitivity factor (ISF) accordingly.^34^ SMB allows the delivery of small boluses and in return reduces the temporary basal rate to safely deliver the peak insulin at the optimal point in time. UAM can be used instead of or in addition to the manual entry of CHO. This feature enables the algorithm to respond to a significant change in BG by calculating an adjusted but safe amount of insulin.^35^ The code is written in the programming languages Java, JavaScript, and Kotlin.

### Implementation of AndroidAPS in MATLAB^®^

The new implementation in MATLAB^®^/Simulink^®^ should not only produce the same results as the original software, but also—where possible—preserve its structure, variable names, and so on. in order to enable error tracing. Porting AndroidAPS (version 2.6.4) to MATLAB^®^ required adaptions in terms of syntax, data types and other aspects. However, it was tried to keep the new code as close to the original as possible, even though this was partly detrimental to efficiency.

### Verification of the AndroidAPS Implementation in MATLAB^®^

Verification of the re-implementation is conducted independently of T1DMS in MATLAB^®^, that is, outside the Simulink^®^ environment. It takes place in two steps. First, the unit tests contained in the OpenAPS code repository (version 0.7.0)^36^ are run. As those only cover the basic functions of the algorithm, additional data was generated in AndroidAPS to define a further set of integration tests and verify the correctness of the entire implementation including the features Autosens, SMB, and UAM. In the scope of these tests several variables computed by the algorithm are collated with the reference values. Among these are administered basal and bolus insulin (insulin on board, IOB), current active CHOs (carbs on board, COB), sensitivity ratio (Autosens) as well as the finally determined basal rate and SMBs.

### Simulation with the Type 1 Diabetes Metabolic Simulator

**Figure 2.**
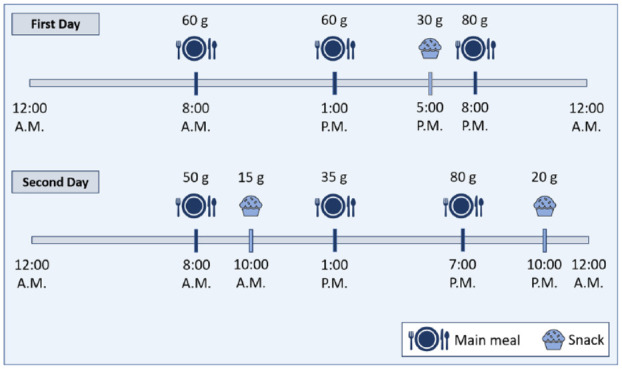
Times of day and amount of CHO of the main meals and snacks given in the two-day-scenario used for the simulation.

After verification of the implementation, a simulation is carried out. It is performed on all 30 in silico patients contained in the T1DMS. Scenario 4 from Toffanin et al.^33^ is used to achieve comparability of the results. The scenario covers two days, starting at midnight. Figure 2 shows the times of day and the amount of CHO consumed for both days. If the measured glucose level falls below 65 mg/dl, a rescue sugar, also called hypotreatment (HT), of 16 g is administered. The minimum interval between two HT is 30 minutes. Meal boluses are delivered 15 minutes before CHO intake. For reasons of comparability, the calculated meal bolus amount is delivered only as a half bolus (SMB_HB), following Toffanin et al.^33^ Autosens, SMB and UAM are activated. The safety parameters “maximum basal rate” and “maximum IOB” are set to 10 U/h and 10 U, respectively.^33^ All simulations are performed on a personal computer with Intel^®^ Core^TM^ i5-8500 CPU and 8 GB RAM using MATLAB^®^ Parallel Computing Toolbox.^37^

### Metrics of Glycemic Control

The simulation results of the glycemic control are evaluated by means of the consensus metrics as described by Danne et al.,^38^ Battelino et al.^39^ and Maahs et al.^40^ To compare our results to those in the literature, we chose the same subset as selected by Toffanin et al.^33^ Mean BG (M), the standard deviation (SD) and the coefficient of variation (CV); furthermore, the percentages of time in target range (TIR), in tight target range (TTT), in level 2 hyperglycemic range (Ta250) and in level 1 hypoglycemic range (T54-70).

Table 1 summarizes the corresponding thresholds of the different glycemic ranges. Also, the average number of HT per patient and the low blood glucose index (LBGI) are determined. These metrics are computed in three blocks: overall (symbol “O,” ie, during day and night), at night (symbol “N,” ie, between midnight and 8:00 A.M.), and post-prandial (symbol “PP,” ie, during periods within 4 hours after a meal). All metrics are presented as median and interquartile range (IQR, 25th to 75th percentiles) as suggested by Danne et al.^38^ Following the recommendation of Danne et al.^38^ and Battelino et al.,^39^ the percentages of time in level 1 hyperglycemic range (T180-250) and level 2 hypoglycemic range (Tb54) as well as the high blood glucose index (HBGI) are also stated.

**Table 1.** Threshold BG values of the different glycemic ranges

Range	Threshold BG values
Level 2 hypoglycemic range	BG ≤ 54 mg/dl
Level 1 hypoglycemic range	54 mg/dl < BG ≤ 70 mg/dl
Target range	70 mg/dl < BG ≤ 180 mg/dl
Tight target range	70 mg/dl < BG ≤ 140 mg/dl
Level 1 hyperglycemic range	180 mg/dl < BG ≤ 250 mg/dl
Level 2 hyperglycemic range	BG > 250 mg/dl

## Results

### Verification

The re-implementation of oref0 in MATLAB^®^ passed all unit tests. The integration tests designed to verify Autosens, UAM, and SMB identified no discrepancies between the results obtained in MATLAB^®^ and the data generated with AndroidAPS.

### Simulation

**Figure 3.**
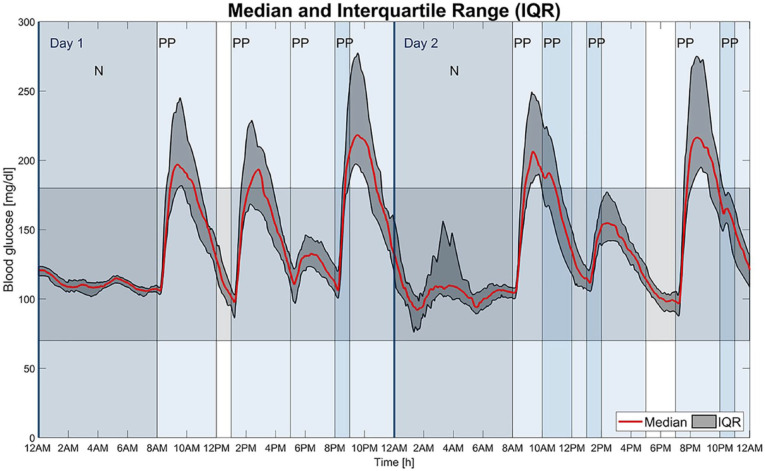
Median and IQR (25th to 75th percentiles) of the BG values of all 30 in silico patients, plotted over the duration of the two-day scenario. The target range is colored as a horizontal area, the postprandial periods (PP) and the two nights (N) as vertical areas.

**Figure 4.**
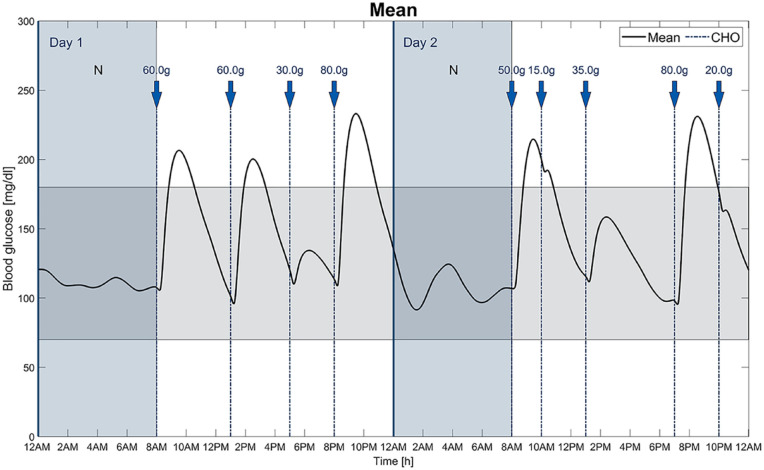
Mean of the BG values and the CHO intake of all 30 in silico patients. The target range is shown as a colored horizontal area and the two nights (N) are marked as colored vertical areas.

**Figure 5.**
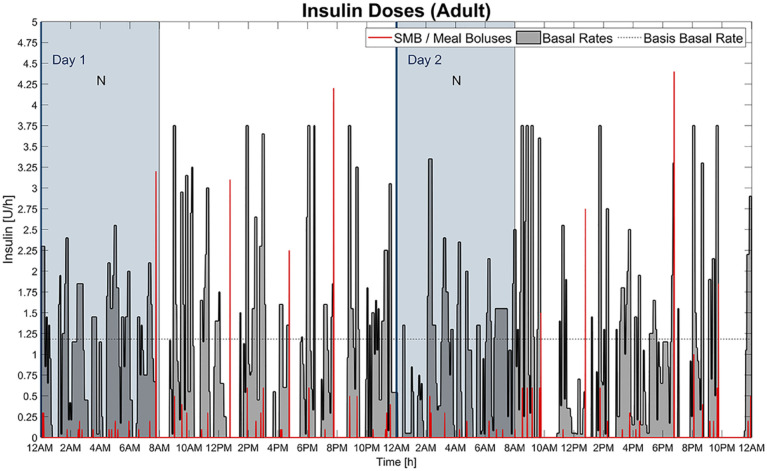
Meal boluses with SMB, basal rates as well as basis basal rate calculated by AndroidAPS during the 48 h simulation, shown as an example for an adult in silico patient. The two nights (N) are marked as colored vertical areas.

The simulation time of the considered two-day scenario for a single virtual patient was approximately three minutes. During simulation of the total population, the 30 virtual patients were distributed to 6 parallel workers by MATLAB^®^’s Parallel Computing Toolbox. Accordingly, the 30 virtual patients were divided into 5 batches and thus simulated within approximately 15 minutes. The results for the metrics described above are listed in Table 2. For comparison, the table also includes the simulation results of Toffanin et al.^33^ Figure 3 visualizes the results, that is, the median and IQR of the BG values of all patients, following the recommendation of Danne et al.^38^ and Battelino et al.^39^ Figure 4 illustrates the mean BG of all patients together with the CHO intake. Table 3 presents the simulation results separated into the three age groups children, adolescents, and adults. Finally, Figure 5 exemplary visualizes the temporary basal rates, the meal boluses and SMB calculated by AndroidAPS for a single virtual patient.

**Table 2.** List of metrics for the performed simulation. Where available, the values from Toffanin et al.^33^ under the name of SMB_HB are given for comparison. All values are stated as median [25th to 75th percentiles], except M_O and M_PP, which are given as mean (±SD) due to the listing in Toffanin et al.^33^

	O	N	PP
M [mg/dl]			
This work	140.38 (±13.02)	108.14 [106.12, 112.28]	160.53 (±20.16)
Toffanin et al.^33^	135.41 (±19.71)	121.34 [112.73, 139.01]	141.33 (±22.66)
SD [mg/dl]			
This work	37.95 [30.85, 52.74]	12.81 [8.03, 19.07]	35.12 [27.50, 48.76]
Toffanin et al.^33^	25.51 [19.76, 31.89]	13.57 [10.53, 17.20]	26.91 [19.91, 33.74]
CV			
This work	0.28 [0.24, 0.35]	0.12 [0.07, 0.17]	0.23 [0.18, 0.27]
Toffanin et al.^33^	0.20 [0.16, 0.23]	0.11 [0.08, 0.13]	0.19 [0.15, 0.25]
TIR [%]			
This work	82.45 [73.72, 90.28]	100.00 [96.99, 100.00]	70.67 [59.93, 85.51]
Toffanin et al.^33^	92.59 [82.56, 98.07]	100.00 [95.06, 100.00]	90.15 [72.77, 97.72]
TTT [%]			
This work	58.21 [52.72, 70.64]	97.66 [90.03, 100.00]	35.26 [26.60, 51.93]
Toffanin et al.^33^	57.25 [38.56, 80.51]	86.09 [64.12, 100.00]	45.10 [27.23, 75.13]
Ta250 [%]			
This work	0.00 [0.00, 6.70]	0.00 [0.00, 0.00]	0.00 [0.00, 10.15]
Toffanin et al.^33^	0.00 [0.00, 0.00]	0.00 [0.00, 0.00]	0.00 [0.00, 0.00]
T54-70 [%]			
This work	0.00 [0.00, 1.01]	0.00 [0.00, 3.01]	0.00 [0.00, 0.00]
Toffanin et al.^33^	0.69 [0.00, 1.68]	0.00 [0.00, 0.00]	0.00 [0.00, 2.21]
# HT			
This work	0.00 [0.00, 1.00]	0.00 [0.00, 1.00]	0.00 [0.00, 0.00]
Toffanin et al.^33^	0.00 [0.00, 4.00]	0.00 [0.00, 0.00]	0.00 [0.00, 3.00]
LBGI			
This work	0.20 [0.13, 0.43]	0.43 [0.30, 0.81]	0.04 [0.02, 0.07]
Toffanin et al.^33^	0.37 [0.07, 1.01]	0.05 [0.00, 0.31]	0.30 [0.04, 1.20]
Additional metrics			
T180-250 [%]	15.93 [9.72, 19.33]	0.00 [0.00, 0.00]	26.23 [14.49, 31.19]
Tb54 [%]	0.00 [0.00, 0.00]	0.00 [0.00, 0.00]	0.00 [0.00, 0.00]
HBGI	3.35 [2.13, 5.38]	0.10 [0.01, 0.44]	5.54 [3.56, 8.56]

**Table 3.** List of metrics for the performed simulation with the results separated into the three age groups children, adolescents, and adults.

	O	N	PP
M [mg/dl]			
Children	151.11 (±8.26)	111.31 [108.10, 113.66]	176.45 (±13.72)
Adolescents	142.75 (±10.86)	108.82 [106.23, 110.69]	165.29 (±15.83)
Adults	127.29 (±6.00)	106.52 [105.40, 108.07]	139.84 (±9.43)
SD [mg/dl]			
Children	51.95 [43.14, 58.90]	18.06 [14.74, 26.11]	48.51 [39.37, 52.68]
Adolescents	35.32 [33.04, 55.22]	13.24 [10.13, 25.93]	31.86 [28.89, 48.76]
Adults	27.82 [21.82, 30.85]	6.90 [5.81, 8.04]	25.44 [20.17, 30.58]
CV			
Children	0.35 [0.30, 0.38]	0.16 [0.14, 0.24]	0.26 [0.24, 0.30]
Adolescents	0.26 [0.24, 0.34]	0.12 [0.10, 0.23]	0.21 [0.19, 0.25]
Adults	0.22 [0.18, 0.24]	0.06 [0.06, 0.07]	0.18 [0.16, 0.22]
TIR [%]			
Children	73.81 [70.98, 76.99]	98.81 [92.94, 100.00]	61.71 [53.64, 66.96]
Adolescents	83.15 [70.98, 85.28]	100.00 [93.35, 100.00]	70.67 [56.37, 75.60]
Adults	93.60 [90.28, 100.00]	100.00 [100.00, 100.00]	90.51 [85.51, 100.00]
TTT [%]			
Children	54.39 [51.30, 57.62]	91.80 [84.84, 95.74]	28.06 [23.33, 35.63]
Adolescents	56.44 [50.82, 61.99]	95.64 [84.11, 99.79]	32.89 [20.35, 37.53]
Adults	72.79 [70.64, 76.26]	100.00 [100.00, 100.00]	57.03 [51.93, 61.93]
Ta250 [%]			
Children	5.41 [0.00, 10.59]	0.00 [0.00, 0.00]	8.20 [0.00, 14.89]
Adolescents	0.00 [0.00, 6.84]	0.00 [0.00, 0.00]	0.00 [0.00, 10.15]
Adults	0.00 [0.00, 0.00]	0.00 [0.00, 0.00]	0.00 [0.00, 0.00]
T180-250 [%]			
Children	19.39 [16.77, 23.12]	0.00 [0.00, 4.05]	30.23 [27.09, 37.04]
Adolescents	16.83 [14.72, 19.23]	0.00 [0.00, 1.04]	27.99 [24.40, 30.75]
Adults	6.40 [0.00, 9.72]	0.00 [0.00, 0.00]	9.49 [0.00, 14.49]
T54-70 [%]			
Children	0.40 [0.00, 1.53]	1.19 [0.00, 4.57]	0.00 [0.00, 0.00]
Adolescents	0.00 [0.00, 1.08]	0.00 [0.00, 3.22]	0.00 [0.00, 0.00]
Adults	0.00 [0.00, 0.00]	0.00 [0.00, 0.00]	0.00 [0.00, 0.00]
Tb54 [%]			
Children	0.00 [0.00, 0.00]	0.00 [0.00, 0.00]	0.00 [0.00, 0.00]
Adolescents	0.00 [0.00, 0.00]	0.00 [0.00, 0.00]	0.00 [0.00, 0.00]
Adults	0.00 [0.00, 0.00]	0.00 [0.00, 0.00]	0.00 [0.00, 0.00]
# HT			
Children	0.00 [0.00, 1.00]	0.00 [0.00, 1.00]	0.00 [0.00, 0.00]
Adolescents	0.00 [0.00, 1.00]	0.00 [0.00, 1.00]	0.00 [0.00, 0.00]
Adults	0.00 [0.00, 0.00]	0.00 [0.00, 0.00]	0.00 [0.00, 0.00]
LBGI			
Children	0.30 [0.21, 0.46]	0.60 [0.50, 1.05]	0.04 [0.02, 0.06]
Adolescents	0.37 [0.07, 1.01]	0.05 [0.00, 0.31]	0.30 [0.04, 1.20]
Adults	0.13 [0.08, 0.15]	0.24 [0.16, 0.30]	0.04 [0.02, 0.07]
HBGI			
Children	4.96 [4.27, 7.09]	0.41 [0.11, 0.75]	7.73 [6.54, 11.25]
Adolescents	3.27 [2.64, 6.48]	0.18 [0.03, 0.75]	5.47 [4.38, 9.84]
Adults	1.60 [1.25, 2.30]	0.01 [0.00, 0.03]	2.52 [2.04, 3.75]

## Discussion

The results show that the new simulation setup keeps the simulation time very low, resulting in a speed-up of 960 compared to real time. In fact, preliminary tests have shown that further code optimization in respect of the strengths of MATLAB^®^ (eg, transformation of for loops into vectorized operations) might account for an additional speed-up factor of 10 or more. However, since reducing the simulation time was not the only criterion for the implementation, this adaptation was deliberately omitted in this work to retain the architecture of the original AndroidAPS code. This should enable the developers of AndroidAPS to comprehend the implementation in MATLAB^®^ as easily as possible.

Table 2 shows that the simulation results of this work differ from those of Toffanin et al. These discrepancies have several causes which are explained in order. Firstly, two different versions of T1DMS are used (the 2014 version in this paper, the 2017 version in Toffanin et al.). Secondly, the T1DMS is based on a stochastic simulation which never produces the same result twice unless the random seed is identical; the seed used by Toffanin et al. is, however, unknown.^33^ Furthermore, not all parameter settings could be extracted from the original paper. Finally, the population size of the present simulation comprises only 30 in silico patients, 10 in each age group, whereas that in Toffanin et al. includes 100 patients of unknown age group membership.^33^ As can be seen from Table 3, however, the values significantly vary depending on the average age of the population. The unknown composition of the virtual population used by Toffanin et al. is therefore another likely source of considerable error.

Accordingly, discrepancies were to be expected and do not necessarily indicate erroneous results. Also, since no time series data of Toffanin et al. is publicly available, no meaningful statistical comparison of both datasets can be performed. The numbers must therefore be interpreted with caution. In fact, some of the employed metrics are highly sensitive to small disturbances and do not reflect deviations of the time series in a linear manner. A comparison of the mean blood glucose (M) values for the three time periods (O, N, PP) yields relative errors of 3.7 %, 10.9 %, and 13.6 %, respectively, which is in the order of magnitude of the differences between the three age groups.

## Conclusion

In this work, the source code of the DIY APS AndroidAPS was re-implemented in MATLAB^®^/Simulink^®^ to conduct in silico trials in the T1DMS. The new approach massively reduces simulation time compared to previous work but introduces a possible error source in the shape of faulty implementation. In fact, a comparison with the values in the literature shows relative errors in the range between 4% and 14% for mean blood glucose. However, differences in the results were to be expected for numerous reasons and do not necessarily indicate an error. What is more, unit tests and integration tests support that the new implementation is sound.

Still, for a distinct quantitative comparison of the data and maximal reliability, simulation should be repeated both in the setup presented by Toffanin et al.^33^ and in the new implementation for an identical virtual population and with identical random seed.

The speed-up of almost 1000 in comparison to real time strongly facilitates even extensive numerical simulations including a large number of virtual patients. This gives developers the opportunity to test new software versions quickly and comprehensively, before they are first tried out in real life. Because the development process is currently not subject of professional or regulatory quality assurance, this may make a decisive contribution to increasing safety of AndroidAPS. In fact, one observation during the in silico study was that hypoglycemia occurred several times. It is now easy to analyze these events and deduce corrections or improvements to the algorithm, if appropriate. Nevertheless, simulation results must always be critically questioned and classified, as simulators such as the T1DMS can never fully represent reality. However, the efficient implementation paves the way for preclinical trials and thus for randomized controlled trials of AndroidAPS, which would be the first step for an approval.
